# Two Foreign Antimicrobial Peptides Expressed in the Chloroplast of *Porphyridium purpureum* Possessed Antibacterial Properties

**DOI:** 10.3390/md20080484

**Published:** 2022-07-28

**Authors:** Subing Han, Jialin Zhao, Ping Liu, Kang Wang, Song Qin, Zhenjun Zhao, Yulin Cui

**Affiliations:** 1 College of Life Sciences, Yantai University, Yantai 264000, China; subinghan@s.ytu.edu.cn (S.H.); xinpingliu@s.ytu.edu.cn (P.L.); zhaozhenjun@ytu.edu.cn (Z.Z.); 2 Key Laboratory of Coastal Biology and Biological Resource Utilization, Yantai Institute of Coastal Zone Research, Chinese Academy of Sciences, Yantai 264003, China; wangkangsdut@126.com (K.W.); sqin@yic.ac.cn (S.Q.); 3 Yantai Marine Economic Research Institute, Yantai 264003, China; zhaojialin@yt.shandong.cn

**Keywords:** *Porphyridium purpureum*, chloroplast transformation system, microparticle bombardment, antimicrobial peptides, NZ2114, Piscidin-4, inhibitory effect

## Abstract

To solve the problem of antibiotic abuse in aquaculture and to utilize the application potential of antimicrobial peptides (AMPs), a chloroplast transformation system of *Porphyridium purpureum* was successfully constructed for effectively expressing two exogenous AMPs. The endogenous fragments of 16S rDNA/*trnA*-23S rDNA were used as flanking fragments for the homologous recombination in the chloroplast genome. Two AMPs encoded by the transformation vector were controlled by the native promoter *psbB* in a polycistron. The plasmids were transferred into *P. purpureum* via particle bombardment and the transformation vectors were screened using phosphinothricin (*bar*), a dominant selection marker under the control of the *psbA* promoter. Subsequently, in the positive transformed colonies, the exogenous fragments were found to be inserted in the flanking fragments directionally as expected and two foreign AMPs were successfully obtained. Finally, two exogenous peptides with antibacterial properties were obtained from the transformed strain. The two AMPs expressed by the transformed strain were shown to have similar inhibitory effects to antibiotics by inhibition tests. This suggested that AMPs can be introduced into aquaculture using baited microalgae, providing new ideas and ways to solve a series of aquaculture diseases caused by bacteria.

## 1. Introduction

Although aquaculture is currently the fastest growing global food industry, it is facing the serious threat of a high mortality of aquatic animals due to infectious diseases caused mainly by bacteria [1,2]. One of the most prominent and effective measures for addressing this threat involves the use of antibiotics, especially broad-spectrum antibiotics, which significantly affect both Gram-positive and Gram-negative bacteria. However, high-yield intensive farming methods cannot avoid the abuse of antibiotics, which leads to the development of bacterial resistance. Disease pandemics caused by antibiotic-resistant bacteria pose serious threats to animal and human health and cause major economic loss [2]. In fact, the European Union already announced a total ban on the use of antibiotics in feed in 2006. Subsequently, the United States, Japan, China, and other countries have introduced various policies to reduce or even ban the use of antibiotics in feed [3]. Vaccinations may be another method that are safer and more effective compared to antibiotics. Nevertheless, the production of vaccines would firstly be subject to a series of regulatory challenges, secondly, the high production cost, transportation cost, and administration cost of vaccines would put unbearable pressure on the aquaculture industry, and thirdly, it was difficult to control the dose and efficacy of vaccines for receptors in different growth periods [4]. A series of biological control strategies have also been explored, yet it has remained difficult to achieve the effect of replacing antibiotics [5]. The pressure of antimicrobial resistance has become a global problem and new antimicrobial drugs must be urgently developed for the prevention and treatment of aquaculture diseases.

Antimicrobial peptides (AMPs) are a class of basic peptide substances with antimicrobial activity, and natural AMPs can be expressed in most organisms. Unlike conventional antibiotics which have selective antibacterial effects, AMPs use multiple mechanisms to eliminate bacterial infections, including direct bactericidal activity and immunomodulatory functions, and plays an important role in both nonspecific and specific immunity. AMPs have multiple modes of action and broad-spectrum antimicrobial properties, rendering the development of drug resistance impossible [6]. Although natural AMPs may have more or less toxic side effects and even more serious hemolytic activity, we are still committed to developing and synthesizing more stable and safer antimicrobial peptides to solve the problem of bacterial drug resistance [7]. NZ2114 is a derivative of Plectasin, a cationic antimicrobial peptide, which shows potent in vitro activity against Gram-positive bacteria, especially *Staphylococcus aureus* and *Streptococcus pneumoniae*, via electrostatic attraction to the negatively charged phospholipids of microbial membranes, leading to membrane disintegration [8]. Compared with the conventional antibiotics, the activity of NZ2114 (0.028–0.9 μmol/L) was stronger than that of ampicillin (1.35~172.50 μmol/L) and vancomycin (0.71~5.67 μmol/L), and also possessed a very long postantibiotic effect (PAE) [8]. Piscidin-4 can bind to the lipopolysaccharide on the outside of the bacterial membrane via an electrostatic gravitational force, disturbing the cell wall in that area and altering its stability, which allows it to cross the area smoothly, come to the proximity of the cell membrane, and eradicate the microbial biofilm. Piscidin-4 was highly active against Gram-negative bacteria, including *Vibrio* spp., that pose a serious threat to aquaculture and possessed immunomodulatory properties [9,10]. Different studies have shown that both AMPs, NZ2114 and Piscidin-4, have weak host toxicity and low hemolytic activity against vertebrates, suggesting that they are more suitable for antibacterial studies [11,12,13]. A point worth stating was that the antibacterial spectrum of NZ2114 and Piscidin-4 complement each other, and if they were recombinantly expressed in the same host, most of the bacteria would be inhibited.

Antibiotics are no longer the first choice for the eradication of pathogenic bacteria in aquaculture due to the continuous development of bacterial resistance and the emergence of issues related to consumer health risks. Among the novel antimicrobial compounds, AMPs are preferred because of their target specificity, rapid mode of action, and lack of bacterial resistance [14]. To utilize the complete potential of AMPs, their manner of application to aquaculture must be optimized. One method involves the direct addition of AMPs to the feed, and studies have shown that this method can significantly reduce the incidence of bacterial diseases and effectively reduce the emergence of drug resistance genes. However, the addition of purified AMPs to the feed considerably increases the cost of aquaculture, while the residual AMPs in the water body may also lead to eutrophication [15]. Another method involves expressing AMPs heterologously, using bacteria or fungi as vectors, and studies have shown that *Escherichia coli* or *Pichia pastoris* can be used to express high concentrations of pure AMPs [16]. However, the exogenous contamination introduced by these hosts cannot be ignored if they are used directly in aquaculture, so AMPs must be isolated and purified; in addition, the procedure of producing highly active AMPs from bacteria or fungi is expensive and low yield, and involves tedious technical operations to obtain [17]. With the continuous development of microalgae baits which can largely reduce the dependence of aquaculture on traditional feeds, the use of genetic engineering to heterologously express AMPs in microalgae and add them directly to feed appears to be feasible [18].

*Porphyridium purpureum* is a unicellular red alga belonging to division Rhodophyta, class Bangiophyceae, order Bangiales, family Porphyridiaceae, and genus *Porphyridium*, which are widely found in moist soil, fresh water, and walls [19]. The algae are also attractive for biotechnological purposes, as they synthesize extracellular polysaccharides, polyunsaturated fatty acids, phycoerythrin, carotenoids, and vitamins, which are potentially valuable for food, medicine, and nutrition [20,21]. Owing to the relatively short production cycle and high yield per unit, *P. purpureum* has a strong competitive advantage in the economy [22]. As a result, it has been widely used as a bait microalga in aquaculture, and it is expected that *P. purpureum* made to express AMPs via genetic engineering may play a more important role in aquaculture [23,24]. Moreover, the existence of multiple exogenous genes in the high-copy plastid genome of each cell may increase the expression of foreign proteins by several folds [25]. As ideal hosts for the expression of foreign genes, the plastids have been successfully used to produce various therapeutic proteins and industrial enzymes. Combining the above advantages, *P. purpureum* can be an ideal host for expressing foreign AMPs in order to carry out the expression and application of AMPs.

In the present study, two exogenous AMPs, NZ2114 and Piscidin-4, were successfully expressed for the first time by the concurrent transformed into *P. purpureum*. The obtained AMPs were tested to have high in vitro antibacterial activity, which is similar to the antibacterial effect of antibiotics. This application of *P. purpureum* will, to some extent, solve the problem of antibiotic abuse in aquaculture and reduce its negative effects.

## 2. Results

### 2.1. Construction of Chloroplast Transformation Vector for Porphyridium purpureum Containing a Polycistron Expressing Two AMP Genes

**Figure 1:**
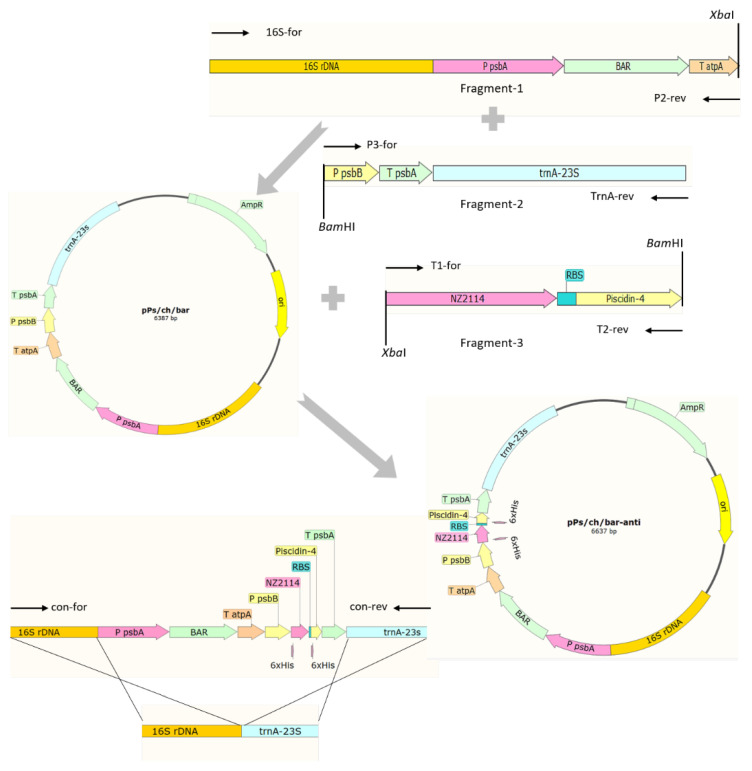
The construction of pPs/ch/bar-anti vector.

The 16S rDNA and *trnA*-23S flanking regions of 992 bp and 927 bp, respectively, as well as the *psbA* promoter (583 bp), *psbB* promoter (205 bp), *atpA* terminator (226 bp), and *psbA* terminator (195 bp), which are endogenous regulatory elements, were obtained via amplification using different primer pairs and the chloroplast genome of *P. purpureum* as the template. The construction of the *P. purpureum* chloroplast transformation vector, pPs/ch/bar-anti, harboring a polycistron expressing two AMPs, is shown in Figure 1. The vector sequence was compared using BLAST, which showed that pPs/ch/bar-anti matched the design of the expression vector (data not shown) and could be used for the next step of transformation.

### 2.2. Two AMPs Were Expressed in the Chloroplast of P. purpureum

After selective culture, few algal colonies were found on the agar plates in which *P. purpureum* was bombarded, while no algal colony was found on the negative control plates. The selection process was as described by Wang et al. (2021) [26], and one strain named M was found to be positive.

**Figure 2:**
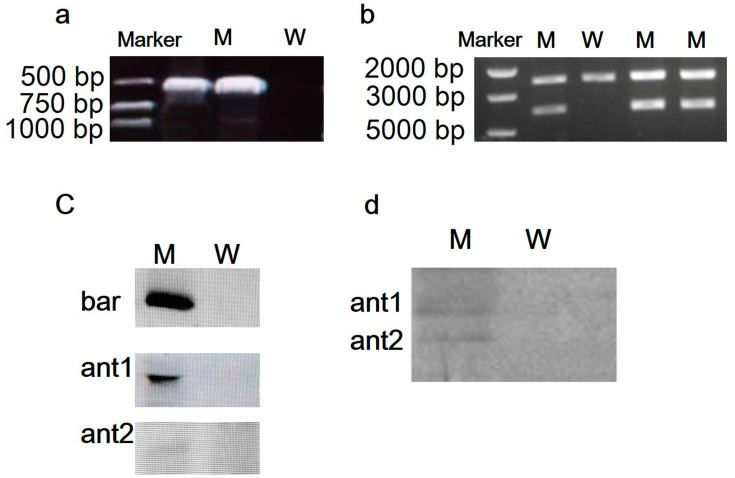
The confirmation of M strain. (**a**) Identification of bar gene in M strain using PCR; (**b**) the detection of complete transformed plasmids in M strain using PCR; (**c**) identification of *bar*, *ant1*, and *ant2* genes in M strain using Southern blotting; (**d**) identification of NZ2114 (ant1) and piscidin-4 (ant2) proteins in M strain using Western blotting. Marker, DNA maker; W, wild *P. purpureum*.

The *bar* gene was amplified using *bar*-specific primers, and large fragments of homology arms and the genes’ expression cassette was cloned using the primer pair con-for/con-rev to identify the integration event in the chloroplast genome in the M strain. A band of approximately 600 bp (Figure 2a), and a single band of 4200 bp (Figure 2b) in the M strain implied the existence of the foreign fragments in the M stain.

In the southern blotting analysis, the transformed colonies showed positive bands (Figure 2c) with digoxin-labeled *bar*, *ant1*, and *ant2* probes, while no band appeared with the wild type *P. purpureum*, demonstrating that the *bar*, *ant1*, *ant2* genes were stably integrated into the *P. purpureum* chloroplast genome. The band of NZ2114 (ant1) 5,3 kDa and the band of Piscidin-4 (ant2) 9.5 kDa were detected in the M strain in Western blotting (Figure 2d), indicating that the two peptides were successfully expressed in the M strain.

### 2.3. The Recombinant AMPs from Crude Protein Extract Possessed Significant Antimicrobial Activity

**Figure 3:**
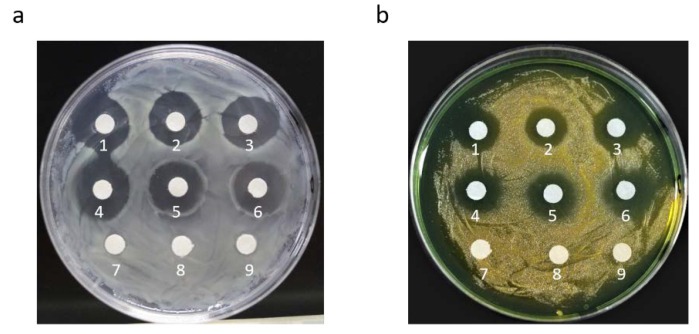
Bacterial inhibitory effect of protein crude extracts containing two AMPs on bacteria compared with antibiotics. (**a**) Antibacterial effect on *S. aureus*; (**b**) antibacterial effect on *V. parahaemolyticus*. For 1–3, 2 μL ampicillin was added; 4–6, 2 μL crude protein extract of the M strain was added; and 7–9, 2 μL sterile water was added.

The antibacterial activity of the recombinant AMPs from the crude protein extracts was evaluated against *Staphylococcus aureus* and *Vibrio parahaemolyticus* using the inhibition circle assay. After 12 h of culture on LB and TCBS solid plates with crude protein extracts and antibiotics, significant inhibition circles were observed (Figure 3). The diameter of the inhibition circles of ampicillin and recombinant AMPs against *S. aureus* and *V. parahaemolyticus* were shown in the Table 1, respectively. To determine the antibacterial activity of antibiotics and recombinant AMPs, we compared their inhibition circle diameters, and the e statistical analysis did not show significant differences (*p* > 0.05) between antibiotics and AMPs.

## 3. Discussion

In this study, the chloroplast expression vector of *Porphyridium purpureum* was successfully constructed via the bombardment transformation method. The growth state of the algal strain was not significantly affected, while the exogenous AMPs were stably expressed and possessed antimicrobial activity.

### 3.1. Chloroplast Expression System Has Significant Advantages

It is generally accepted that a lack of homology in the flanking sequences may result in lower recombination rates [27]. In this study, 16S rDNA/*trnA*-23S was selected as the homologous insertion site, which is widely used for gene insertion in chloroplast transformation, such as in *Haematococcus pluvialis* [28], *Tisochrysis lutea* [29], *Platymonas Subcordiformis* [30], *Momordica charantia L* [31]. Some studies have shown that *trnI*/*trnA* is an effective gene insertion locus in the chloroplast genome [32].

In previous plasmid transformation studies, a range of selectable marker genes have been developed. Furthermore, herbicides have been shown to act on specific processes in the plastid, and therefore, tolerance to herbicides can also be used as a chloroplast selection marker. DL-phosphinothricin is the active ingredient of the herbicide Basta, and the bacterial *bar* gene encodes phosphinothricin acetyltransferase, conferring tolerance to glufosinate or its ammonium salt [29,33]. Hence, the bar gene is a suitable selection marker for *P. purpureum*.

Selection of an appropriate transformation method is essential for genetic transformation. Currently, vectors can be delivered into the chloroplast via various methods, such as the glass bead method and electroshock transformation method. Although bombardment causes significant damage to treated cells, it remains the most efficient method for chloroplast transformation. Here, the newly constructed chloroplast expression vectors were transferred into *P. purpureum* chloroplasts using an optimized bombardment method, allowing the growth of glufosinate-resistant colonies.

The effective expression of foreign genes in algal chloroplasts is usually better obtained when endogenous regulatory elements are used. P*psbA* is a commonly used endogenous regulator, which encodes the D1 protein of the photosystem II reaction center, one of the most highly expressed proteins in the plastid [34]. The promoter and 5′ UTR of *psbA* support the highest levels of heterologous protein accumulation [35,36,37], and the −35 element is essential for the initiation of transcription at the *psbA* promoter in tobacco. In this study, the −35 element in the *psbA* promoter is predicted to play an important role. In addition, the exogenous promoter showed a reduction in foreign protein expression compared to that observed with the endogenous *psbA* promoter in tobacco [38]. Hence, the utilization of endogenous regulatory elements achieved efficient chloroplast transformation. Furthermore, the *psbB* promoter, reports on which are few, was selected as the other promoter. Thus, this study provides a new promoter for gene transformation research. However, the efficiency of the *psbA* and *psbB* promoters in *P. purpureum* warrants further investigations.

The current chloroplast expression system still faces multiple challenges: (1) identified a more economical, simple, and reliable mechanism to allow the accumulation of exogenous gene expression in chloroplasts; (2) optimized multiple exogenous gene co-expression strategies [39]. Therefore, the chloroplast expression system still needs further optimization to be better put into application.

### 3.2. Microalgae Can Serve as Good Protein Bioreactors

Currently, it is mainly the *Escherichia coli* and *Pichia pastoris* systems that are used for expressing AMPs. There is no doubt that the use of *E. coli* as a synthesizer of AMPs is simple and easy because its genome has been well documented. However, the process of expressing the target gene in *E. coli* lacked processing modifications, which will lead to the low biological activity of the target protein [40]. Although the *P. pastoris* system has a better gene expression regulatory mechanism and can perform some post-translational modifications, its fermentation cycle was long, it did not utilize the expression of exogenous genes, and it was easily contaminated during the fermentation process. Most importantly, the AMPs expressed in *P. pastoris* cannot be applied directly, yet the antimicrobial activity of the isolated and purified AMPs would be compromised to some extent [41].

On the contrary, microalgae have many advantages as bioreactors for heterologous protein production. First, microalgae are capable of post-translational modifications that allow the production of complex eukaryotic proteins. Second, although microalgae are eukaryotes, they have a short growth cycle and are economically efficient. Importantly, microalgae are free from human and animal pathogens and endotoxins, and the microalgae cells which produce antimicrobial peptides can be directly applied and are considered safe food [42]. Therefore, there will be enormous potential for the application of *P. purpureum* capable of producing AMPs in aquaculture.

### 3.3. Expression of Recombinant AMPs in P. purpureum Has Excellent Application Prospects

So far, some unicellular algae have been reported to successfully express AMPs, such as Chlorella vulgaris, Haematococcus pluvialis, and Chlamydomonas reinhardtii [26,28,43]. We have previously expressed Piscidin-4 and NZ2114 in Chlorella vulgaris without harmful effects on transplastomic plants. However, no relevant antibacterial experiments have been performed [26]. Scholars have successfully expressed VP28 in C. reinhardtii and tested its efficacy with herring infected with the white spot syndrome virus (WSSV). The survival rate of shrimp fed transgenic C. reinhardtii was significantly higher than that fed the wild type [44]. This shows that the antibacterial effect of antimicrobial peptides can still be achieved by directly feeding the algae expressing antimicrobial peptide genes, and it can also provide abundant nutrients. We have successfully expressed two AMPs in P. purpureum and performed antibacterial experiments. The co-expression of the two peptides in P. purpureum elicited obvious antibacterial effects and may be further studied for antibacterial breeding experiments with bait microalgae. This will reduce the usage of antibiotics in aquaculture.

### 3.4. Protein Expression Still Need to Be Improved

Although we have successfully expressed NZ2114 and Piscidin-4 in *P. purpureum*, the protein expression level compared to *P. pastoris* (1309 mg/L) was relatively low [41]. The expression level of recombinant proteins is affected by various factors such as the nature of the gene itself, expression vector, host bacteria, and culture conditions. Therefore, we can improve the expression of foreign genes by optimizing their sequence, designing efficient chloroplast expression vectors, and optimizing the 5′-terminal regulatory sequences to improve recombinant protein expression. The selection and utilization of efficient regulatory sequences and appropriate combinations can effectively achieve the high expression of recombinant proteins. The base composition and codons of chloroplast genomes vary with plants. Therefore, the synthesis of foreign genes must be optimized according to the codon preference of the chloroplast genome. In addition, we can also use the method developed in this study to fuse and express several proteins with the aim of improving protein expression.

## 4. Materials and Methods

### 4.1. Strain and Growth Conditions

*Porphyridium purpureum* (K.M.Drew and R.Ross 1965) from Freshwater Algae Culture Collection at the Institute of Hydrobiology, Chinese Academy of Sciences, was cultured in artificial sea water (f/2) liquid medium [45] at 25 °C under constant illumination of 80–90 μmol photons m^−2^ s^−1^ in a GTOP-illuminated incubator (Tuopu, China). *Escherichia coli* DH5α was grown in Luria–Bertani (LB) medium, *Vibrio parahaemolyticus* in TCBS medium, and *Staphylococcus aureus* in LB solid medium, in a DHP-9052 electric thermostat incubator (Yiheng, China) at 37 °C.

### 4.2. Construction of the Chloroplast Expression Vector

Approximately 200 mL *P. purpureum* in late-logarithmic growth phase was collected via centrifugation at 10,000× g for 5 min, and the genomic DNA was extracted using the plant genomic DNA extraction kit (Accurate Biotechnology, Dalian City, China). Different primers were designed to separately amplify the endogenous psbA promoter (P1-for/P1-rev), atpA terminator (P2-for/P2-rev), psbB promoter (P3-for/P3-rev), and psbA terminator (P4-for/P4-rev) as well as the 16S rDNA (16S-for/16S-rec) and trnA-23S (TrnA-for/TrnA-rev) flanking regions of *P. purpureum* (Table 2). Same concentrations of polymerase chain reaction (PCR) products covering the 16S rDNA, PpsbA, bar, TatpA and PpsbB, TpsbA trnA-23S rDNA were mixed (100–200 ng/μL) as templates, followed by fusion PCR to obtain fragment-1 and fragment-2; then, the PCR products were ligated with the pMD18T vector (TaKaRa, China) using the original TA cloning kit to produce vector pPs/ch/bar. All fragments were sequenced by the Sangon Biotech Co, Ltd. (Shanghai, China).

The genes encoding two exogenous AMPs, NZ2114 (GenBank No. 6K50_A) and Piscidin-4 (GenBank No. AKA60777.2), were codon-optimized and synthesized by Ribo (China) based on the published gene sequences, and named *ant1* and *ant2*. Primer pairs T1-for/T1-rev and T2-for/T2-rev were designed with appropriate restriction enzyme cut sites and ribosome-binding sites (RBS) for amplification of *ant1* and *ant2*, respectively. All fragments were sequenced by Sangon Biotechnology Co. (Shanghai, China). After ligation of *ant1* and *ant2* using fusion PCR, the products were ligated with the *Xba*I/*Bam*HI-digested pPs/ch/bar vector to generate the pPs/ch/bar-anti vector.

### 4.3. Biolistic Transformation and Selection of Transformed Strains

In total, 100 mL logarithmic growth phase algal fluid was taken and the cells were collected via centrifugation at 6000 rpm for 5 min. The cell density was adjusted to 1.5–2 × 10^8^ cells/mL and 200 μL cell suspension was spread on the center of solid medium to form a circle 2 cm in diameter. Plasmid DNA (5 μg in 5 µL) was coated onto gold carrier particles according to a previously published method by Jiang et al. (2002) [46]. To successfully obtain transformed *P. purpureum*, 9 cm flight distance and 650 psi acceleration pressure were used. Cells transformed with the empty vector plasmid were used as the negative control.

Solid plates cultured with bombarded algal cells were placed in a recovery culture at 25 °C for 8 h under dark conditions, after which they were transferred to f/2 liquid medium for 40 h under normal conditions to restore cell viability. After recovery, the transformed cells were subjected to a series of screens using f/2 agar plates containing different concentrations of glufosinate, as described previously [26]. Finally, 40 single positive algae clones were randomly selected and cultured in liquid f/2 selection medium containing 5 μg mL^−1^ phosphinothricin for subsequent experiments.

### 4.4. Molecular Identification of Transformed Strains

The selected positive clones were cultured for 20 days, and the algal fluid from the post-logarithmic phase were collected after centrifugation at 7000 rpm for 5 min. Whole genomic DNA was extracted for performing PCR analysis, and wild type *P. purpureum* DNA was used as the control. First, the bar gene was examined using bar-for and bar-rev primers, and mutant strains carrying the bar gene were selected to amplify the upstream and downstream flanking fragments of the expressing cassette using specific primers (con-for/con-rev). The colonies that tested positive in PCR were finally selected for sequencing, and the transformed strains with correct sequences were cultured in the selection medium for subsequent analysis.

Transformed strains with correct sequences were selected and used for Southern blotting using DIG High Prime DNA labeling and detection starter I kit (Roche, Munich Germany). DIG probes were prepared using the *bar*, *ant1*, and *ant2* genes as templates to determine whether the exogenous fragments were stably inserted into the chloroplast genome.

The transformed strains that showed positive results in Southern blotting were cultured and 3L of algal cells in late-logarithmic growth phase were collected and resuspended in 10 mL phosphate buffered saline (PBS) after being ground in liquid nitrogen. The Western blotting analysis was performed according to the method mentioned by Wang et al. (2020) [28]. The expressed proteins were identified using mouse anti-His IgG (Absin, Shanghai, China) and goat anti-mouse IgG (Absin, Shanghai, China) conjugated to horseradish peroxidase (HRP) (Sigma, USA) to determine if the exogenous proteins can be expressed.

### 4.5. Determination of Antibacterial Activity of Recombinant AMPs in Crude Protein Extracts

The transformed strains that showed positive results in Western blotting were cultured and the crude protein was extracted using 5 L of algal cells in late-logarithmic growth phase to resuspend in 2 mL phosphate buffered saline (PBS) after grinding in liquid nitrogen [28]. The antibacterial activity of the protein extracts against Gram-positive bacteria (*V. parahaemolyticus* and *S. aureus*) was analyzed using the inhibition circle method [47]. A single bacterial colony was removed from the sterile culture of each strain and incubated in LB and 2216E medium at 37 °C, 200 rpm for 12 h. One hundred microliters of bacterial suspension (1 × 10^8^ CFU/mL) were added dropwise to the LB and TCBS solid plate and spread evenly with a smear stick. Nine sterile filter paper sheets 6 mm in diameter were placed on the plates; 2 μL ampicillin was applied dropwise to three plates, while 2 μL crude protein extract of the M strain were added to three plates, and 2 μL sterile water to the remaining three plates, followed by incubation at 37 °C for 12 h. The diameters of the inhibition circles were recorded with a digital camera and measured.

## 5. Conclusions

In this research, two exogenous AMPs were successfully and stably expressed without affecting the normal growth of *Porphyridium Purpureum* and were confirmed to have antimicrobial properties. Although the current expression of recombinant AMPs is not sufficient to completely replace antibiotics for the purpose of disease treatment, if aquatic animals were fed with the mutant strain of *P. purpureum* at the early stage of culture, based on the immunomodulatory function of the AMPs themselves, it could theoretically increase the resistance of the aquatic animals themselves to prevent disease pandemics caused by bacterial infections.

## Figures and Tables

**Table 1:** The diameter of the inhibition circles of antibiotic-ampicillin and recombinant AMPs against *S. aureus* and *V. parahaemolyticus*.

	Diameter of the Inhibition Circles of Antibiotic (mm)	Diameter of the Inhibition Circles of AMPs (mm)
*Staphylococcus aureus*	17.03 ± 0.03	17.52 ± 0.34
*Vibrio parahaemolyticus*	14.88 ± 0.74	15.96 ± 0.45

The experiments were performed in biological triplicates (*n* = 3) to ensure reproducibility. All values were presented as means ± SD.

**Table 2:** Primers used for the chloroplast vectors.

Primers	Sequences (5′-3′)	Special Sequences	Target Gene
16S-for	TATCCGGAATCACTGGGCGTAA		16S rDNA
16S-rev	TTTAAGGAGGTGATCCAGCCGC		16S rDNA
TrnA-for	AAGGGGATATAGCTCAGTTGG		*trnA*-23S rDNA
TrnA-rev	GGAGAACCAGCTAGCTCCGGAT		*trnA*-23S rDNA
PpsbA-for	CATCAACTTTATCTAAAGACGA		*psbA* promoter
PpsbA-rev	AATTTTTTTGTTAAATAAAAGTTTTTTGTG		*psbA* promoter
PpsbB-for	CTGTATTGTAGTTTTTTTTAATA		*psbB* promoter
PpsbB-rev	TAATTACTACAATTAGAATTAAACTC		*psbB* promoter
TatpA-for	TCAATAATTAATATTTATAGTGTTCA		*atpA* terminator
TatpA-rev	CTGCTATTTTACTTATCACTCATTA		*atpA* terminator
TpsbA-for	GATTTATAAAAAACAAAAAAGCACTTC		*psbA* terminator
TpsbA-rev	ACTAGGTGTCCCTATTATTGGTATG		*psbA* terminator
bar-for	ATGAGCCCAGAACGACGCC		*bar*
bar-rev	TCATCAAATCTCGGTGACGGG		*bar*
P1-for	*GGCTGGATCACCTCCTTAAA*CATCAACTTTATCTAAAGACGA		*psbA* promoter
P1-rev	*GGCGTCGTTCTGGGCTCATG*AATTTTTTTGTTAAATAAAAGTTTTTTGTG		*psbA* promoter
P2-for	*CCCGTCACCGAGATTTGATGA*TCAATAATTAATATTTATAGTGTTCA		*atpA* terminator
P2-rev	*TCTAGAGGTATTAAAAAAAACTACAATACAG*TCAATAATTAATATTTATAGTGTTCA	*Xba*I	*atpA* terminator
P3-for	*CGAGCTC*GATTTATAAAAAACAAAAAAGCACTTC		*psbB* promoter
P3-rev	*CCAACTGAGCTATATCCCCTT*ACTAGGTGTCCCTATTATTGGTAT		*psbB* promoter
P4-for	AAGGGGATATAGCTCAGTTGG		*psbA* terminator
P4-rev	*GGAATCC*GGAGAACCAGCTAGCTCCGGAT		*psbA* terminator
T1-for	*CCTCTAGA*ATGCATCATCACCATCACCATGGTTTCGGTTGCAACGGTCCCTGG	*Xba*I, 6 × His	*ant1*
T1-rev	TTAGTAGCACTTGCAGACGAAAATAAATTATCCTTATGAAATGGTGATGGTGATGGTGCAT	RBS	*ant1*
T2-for	ATGCACCATCACCATCACCATTTCTTCTTCCACATCATCAAGGG	6 × His	*ant2*
T2-rev	*GGGATCC*TTACTTCCAGACGAGACCGTGGAT	*Bam*HI	*ant2*
con-F for	AGCATCGGCTAACTCCGTGC		16S rDNA-*trnA*-23S
con-F rev	TAACCGCTGCGCCTCAACGC		16S rDNA-*trnA*-23S

Letters underlined correspond to introduced restriction sites or histidine tag; homologous sequences added on the side of the gene are in italics.

## Data Availability

Not applicable.
